# Cryptic Biodiversity and the Origins of Pest Status Revealed in the Macrogenome of *Simulium colombaschense *(Diptera: Simuliidae), History’s Most Destructive Black Fly

**DOI:** 10.1371/journal.pone.0147673

**Published:** 2016-01-25

**Authors:** Peter H. Adler, Tatiana Kúdelová, Matúš Kúdela, Gunther Seitz, Aleksandra Ignjatović-Ćupina

**Affiliations:** 1 Department of Agricultural and Environmental Sciences, Clemson University, Clemson, SC, United States of America; 2 Department of Zoology, Comenius University, Bratislava, Slovakia; 3 District Government of Lower Bavaria, Landshut, Germany; 4 Department for Environmental and Plant Protection, Faculty of Agriculture, University of Novi Sad, Novi Sad, Serbia; Virginia Tech, UNITED STATES

## Abstract

The European black fly *Simulium* (*Simulium*) *colombaschense* (Scopoli), once responsible for as many as 22,000 livestock deaths per year, is chromosomally mapped, permitting its evolutionary relationships and pest drivers to be inferred. The species is 12 fixed inversions removed from the standard sequence of the subgenus *Simulium*. Three of these fixed inversions, 38 autosomal polymorphisms, and a complex set of 12 X and 6 Y chromosomes in 29 zygotic combinations uniquely characterize *S*. *colombaschense* and reveal 5 cytoforms: ‘A’ in the Danube watershed, ‘B’ in Italy’s Adige River, ‘C’ in the Aliakmonas River of Greece, ‘D’ in the Aoös drainage in Greece, and ‘E’ in the Belá River of Slovakia. ‘C’ and ‘D’ are reproductively isolated from one another, and ‘B’ is considered a cytotype of ‘A,’ the probable name bearer of *colombaschense*. The species status of ‘E’ cannot be determined without additional collections. Three derived polytene sequences, based on outgroup comparisons, place *S*. *colombaschense* in a clade of species composed of the *S*. *jenningsi*, *S*. *malyschevi*, and *S*. *reptans* species groups. Only cytoforms ‘A’ and ‘B’ are pests. Within the Simuliidae, pest status is reached through one of two principal pathways, both of which promote the production of large populations of blood-seeking flies: (1) colonization of the world’s largest rivers (habitat specialization) or (2) colonization of multiple habitat types (habitat generalization). Evolutionary acquisition of the ability to colonize large rivers by an ancestor of the *S*. *jenningsi*-*malyschevi*-*reptans* clade set the scene for the pest status of *S*. *colombaschense* and other big-river members of the clade. In an ironic twist, the macrogenome of *S*. *colombaschense* reveals that the name associated with history’s worst simuliid pest represents a complex of species, two or more of which are nonpests potentially vulnerable to loss of their limited habitat.

## Introduction

Pest status is not an inherent biological property, but rather the result of human interests conflicting with organismal traits. The drivers of pest problems, from gene to environment, are not well understood, and the evolutionary innovations and underlying genetic basis that inadvertently set the scene for pest problems are not always clear [1]. For black flies—among the most virulent pests of birds and mammals [2]—the extrinsic factors associated with pest status have been more accessible than the intrinsic, or genetic, factors. Extrinsic factors that promote pest problems of black flies include rainfall, which can increase available breeding areas, and habitat modifications (e.g., impoundment of rivers), which can increase freshwater productivity [3, 4]. Geographic variation in pest status, while initially inexplicable, can sometimes be explained by the presence of cryptic species that differ in blood hosts, breeding habitats, and other organismal properties [5].

Black flies are among the few insects that have routinely killed animals through direct attacks, typically by exsanguination or toxic shock—simuliotoxicosis—from injected salivary constituents [6]. About seven species in the world, all developing in large rivers, have been responsible for large-scale livestock deaths [7]. Though now largely a historical phenomenon, the greatest wholesale slaughter of domestic animals was caused by *Simulium colombaschense* (Scopoli), responsible in some years for more than 22,000 deaths along the Danube River of southeastern Europe [8].

*Simulium colombaschense* was considered “among the most dangerous species in the world,” killing animals over a broad swath beyond the Danube River [9]. The species had caused problems along the Danube for centuries, spawning myths among local people desperate to explain the outbreaks [10, 11]. A popular legend held that the flies arose from a cave in a wall of the river’s gorge where a dragon wounded by St. George had sought refuge and eventually died, spewing the flies from its head. So powerful was the legend that the Austro-Hungarian government ordered two caves in the gorge closed with lead and cement [10, 11]. More pragmatically, the flies were the impetus for one of the first biological studies of black flies [12], ordered in the early 1790s by Maria Theresa, Holy Roman Empress of the Habsburg Monarchy. The last major outbreak of *S*. *colombaschense* occurred in 1950, killing more than 800 animals [9]. Construction of a hydroelectric dam in the 1960s unintentionally eliminated the pest problem by inundating the Danube’s rocky breeding grounds at the Iron Gate, a gorge between Romania and Serbia [9].

The only attacks peripheral to the Danube Basin arose from northeastern Italy’s Adige River and the Biffi canal that runs parallel to the Adige and collects water from it [13]. After construction of the canal, which putatively favored larval development, the flies periodically reduced milk production and killed pastured livestock [14–16].

Linnaeus named this destructive species *Culex lanio* in 1771, but that name was overlooked and the current name (originally as *Oestrus columbacensis*) was applied by Scopoli in 1780, based on an alternative spelling for an old town (Golubac) on the Danube downriver from Belgrade where the species was particularly virulent [17, 18]. The vernacular name “Golubatz fly,” in reference to this town, traces its origins to 1781 [2]. Given the veterinary prominence of *S*. *colombaschense*, it is perhaps appropriately the type species for the genus *Simulium* [18], the largest genus in the family, with 1,767 (81%) of the world’s described species [19].

*Simulium colombaschense* has been placed in the *Simulium reptans* species group based on structural characters [20] now known to overlap with those used to define the *S*. *malyschevi* species group [21]. Toward clarification of the systematics of this nomenclaturally and veterinarily important species, we map its polytene chromosomes and then use the macrogenomic information to prospect for hidden diversity, interpret the taxonomy, resolve evolutionary relationships, and gain insights into the drivers of its historic pest status and the pest status of black flies more generally. By identifying the drivers of pest status, we provide a basis for proactive pest management of black flies, focused on predicting and preventing problems.

## Materials and Methods

### Ethics Statement

All collections of larvae and pupae were made on public land with access from public roads. No specific permissions were required to access sites or collect material, and the collections did not involve endangered or protected species.

### Collection and Preparation of Material

**Fig 1:**
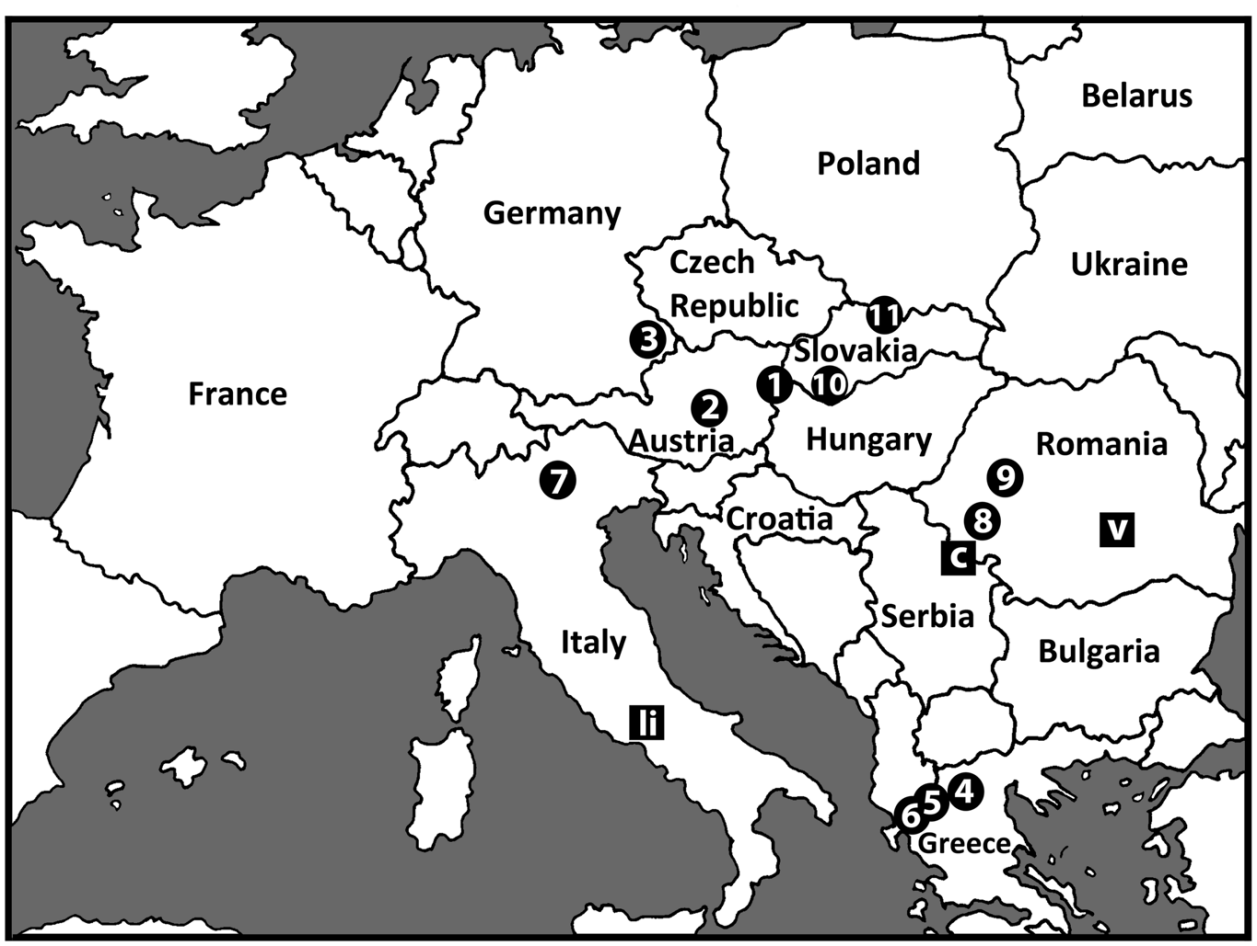
Sampling sites for larvae of *Simulium colombaschense* used in chromosomal analysis. Numbers correspond with collecting information in Table 1. Type localities are indicated for *S*. *colombaschense* (c) at the Iron Gate, *S*. *liriense* (li), and *S*. *voilense* (v).

Larvae of *S*. *colombaschense* were collected by hand from 11 sites in 6 countries, mainly during the spring (April–June) but also in the fall (October) (Table 1, Fig 1), probably representing two generations. They were fixed in modified Carnoy’s solution (1:3 acetic ethanol) and held at -4°C until processing.

**Table 1:** Collection data for chromosomally analyzed larvae of *Simulium colombaschense*.

Site	Country	Location	Altitude (m)	Latitude Longitude	Date	Larvae analyzed	Cytoform
1	AUSTRIA	Stopfenreuth, Danube River	138	48°08'42"N 16°54'10"E	17 April 2011	4	A
2	AUSTRIA	Styria, Gesäuse, Enns River	602	47°34'58"N 14°35'20"E	11 June 2010	2	A
3	GERMANY	Bavaria, Passau, Inn River	301	48°33'17"N 13°26'07"E	28 April 2011	26	A
					24 October 2011	26	A
4	GREECE	Aliakmonas, Aliakmonas (= Haliacmon) River	550	40°18'10"N 21°25'59"E	3 May 2013	34	C
5	GREECE	Melissopetra, Aoös River	355	40°03'23"N 20°37'35"E	6 May 2013	35	D
6	GREECE	Lagkáda, Sarantáporos River	630	40°13'32"N20°50'44"E	4 May 2013	9	D
7	ITALY	Borghetto, Adige River	125	45°42'05"N 10°55'40"E	26 April 2011	11	B
8	ROMANIA	Bârza, Cerna River	110	44°50'26"N22°23'15"E	11 May 2011	1	A
					9 May 2015	2	A
9	ROMANIA	Turdaş, Mureş River	193	45°50'31"N23°07'06"E	9 May 2015	5	A
10	SLOVAKIA	Číčov, Danube River	109	47°45'00"N17°45'47"E	11 April 2011	7	A
11	SLOVAKIA	Liptovský Hrádok, Belá River	640	49°02'28"N19°42'58"E	16 June 2009	1	E

The posterior half of each larva was severed, opened ventrally with fine needles, and Feulgen stained [4, 22], with treatment in cold 5N hydrochloric acid [23]. Larval silk glands with their stained polytene chromosomes, plus one gonad, were transferred to a drop of 50% acetic acid and flattened under a coverslip by applying thumb pressure. Gender was determined by gonadal shape (elongated in females, spherical in males) and confirmed cytologically by the presence of sporadically distributed (female) or clustered (male) meiotic figures.

### Chromosomal Mapping and Analyses

Selected high-quality chromosome preparations from larvae collected in the Inn River, Germany, unless otherwise stated, were photographed under oil immersion on an Olympus BX40 compound microscope. Photographic negatives were scanned with a Nikon Coolscan V and imported into Adobe^®^ PhotoShop^®^ Elements 8 to construct chromosomal maps. Larval carcasses and photographic negatives of chromosomes were deposited in the Clemson University Arthropod Collection, Clemson, SC.

Sections 1–100 of the 3 chromosomes (I, II, III) were numbered on the short (S) and long (L) arms of our maps, according to the *Simulium* subgeneric standard for the IS, IL, IIL, and IIIS arms [24] and the IIS and IIIL arms [25, 26]. Chromosomal landmarks were labeled in accord with those of the subgeneric standard [24]. Banding patterns of stained polytenes of *S*. *colombaschense* were compared with those of the subgeneric standard sequence. Inversions fixed across all populations (i.e., interspecific inversions) are italicized in the text and underlined on the maps, whereas floating (i.e., polymorphic) inversions, and any inversions linked to sex, are in Roman type. We used the same numbers for the same inversions found in previously studied, related species; thus, inversions *IL-1*, *IL-2*, *IIS-1*, *IIL-1*, *IIL-2*, *IIIS-1*, *IIIL-1*, and *IIIL-3* of *S*. *colombaschense* are identical to those identified previously in related species [21]. We used new, consecutive numbers for novel inversions, and we named each heteroband (hb) by the section in which it occurs (e.g., 6hb).

Autosomal polymorphisms with frequencies of 50% or greater in collections of more than 25 larvae were tested for Hardy-Weinberg equilibrium. All other autosomal polymorphisms were too infrequent for meaningful analysis, occurring in fewer than 10 homologues (typically fewer than 5) per inversion per site.

All rearrangements are indicated by brackets or arrows on the photographic maps, dashed if linked to the X chromosome, dotted if linked to the Y, and solid if fixed or autosomally polymorphic. We identified the sex chromosomes of *S*. *colombaschense* by rearrangements (e.g., inversions, heterobands), linked to gender, on one of the three chromosomes (I, II, or III) [27–30]; these rearrangements were expressed homozygously in females (XX) and heterozygously in males (XY). If no rearrangements were linked to the X or Y, we considered the sex chromosomes to be cytologically (i.e., microscopically) undifferentiated (X_0_Y_0_). We prepared idiograms to graphically summarize the sex chromosomes and all other diagnostic rearrangements of *S*. *colombaschense*.

### Terminology

We use the term ‘cytoform’ as a general descriptor for chromosomally distinct entities that can be recognized at an individual or a population level. The term carries no implications as to whether the entities are interbreeding (cytotypes) or reproductively isolated (cytospecies). ‘Pest’ is defined here as a species that in some part of its range has caused economic losses or has been a target for management.

### Phylogenetic Inference

To determine evolutionarily derived chromosomal sequences, we first resolved all rearrangements of *S*. *colombaschense* relative to the *Simulium* subgeneric standard. We used the outgroup-based decisions of Adler & Huang [21], who recognized *IIL-1*, *IIL-2*, *IIIS-1*, and *IIIL-2* as synapomorphies for the *S*. *jenningsi*, *S*. *malyschevi*, and *S*. *reptans* groups, including *S*. *colombaschense*. We used the same outgroups as Adler & Huang [21]—*S*. (*Boophthora*) *erythrocephalum* (De Geer) and *S*. (*Psilozia*) *vittatum* Zetterstedt—to determine the evolutionary polarity of sequences in *S*. *colombaschense*. The designations for the IS and IL arms in the subgenus *Psilozia* are reversed on the original maps [31] that we used for comparison. Only rearrangements that could be resolved in the outgroups were included in phylogenetic inference.

## Results

The chromosomes of 163 larvae (96 females, 67 males) of 167 that we prepared, including 25 parasitized with unidentified mermithid nematodes, were analyzed completely, band by band, against the *Simulium* subgeneric standard. The primary nucleolar organizer was in the subgeneric standard position in IIIL. A chromocenter and B (supernumerary) chromosomes were lacking. The total number of rearrangements in the polytene complement, relative to the subgeneric standard, included 59 inversions (12 fixed, 30 floating, and 17 sex-linked; Tables 2 and 3), 10 elaborated bands (e.g., heterobands), 1 secondary nucleolar organizer, 1 transposed nucleolar organizer, and 1 centromeric dimorphism. More than one-quarter of all inversions were sex-linked, all in the distal half of IS, typically atop interspecific inversion *IS-2*.

**Table 2:** Frequency of inverted constituents for all polymorphic autosomal inversions in larval samples of *Simulium colombaschense*.

	Site 1	Site 2	Site 3 April	Site 3 October	Site 4	Site 5	Site 6	Site 7	Site 8	Site 9	Site 10	Site 11
Cytoform	A	A	A	A	C	D	D	B	A	A	A	E
Females:Males	1:3^1^	1:1	13:13^1^	13:13^1^	23:11	21:14^1^	4:5	5:6^1^	3:0^2^	5:0^1^	5:2	1:0
IS-1^3^					0.43^4^							
IS-4					0.43^4^							
IL-5	1.00	1.00	1.00	1.00				0.95	1.00	1.00	1.00	
IL-8											0.07	
IL-9				0.02								
IL-10	0.12	0.25	0.08	0.10								
IL-11			0.02									
IL-12			0.02									
IL-13	0.12											
IL-14											0.07	
IL-15									0.17			
IL-16							0.06					
IIS-2											0.07	
IIS-3						0.01						
IIL-12	0.12	0.25	0.04	0.08				0.41			0.07	
IIL-13			0.02	0.04					1.00	0.90	0.21	
IIL-14	0.75	0.50	0.63^5^	0.71^5^				0.05			0.57	
IIL-15					0.01							
IIIS-2								0.05				
IIIL-4	0.75	1.00	0.75^5^	0.50^5^	*^6^	1.00	1.00	0.23			0.50	
IIIL-6			0.06	0.06								
IIIL-7			0.02									
IIIL-8				0.02								
IIIL-9	0.12											
IIIL-10											0.07	
IIIL-11												1.00
IIIL-12						0.01						
IIIL-14							0.06					
IIIL-15									0.33			
Mean no. of heterozygous inversions/larva	2.00	1.00	1.46	1.38	0.59	0.06	0.22	1.36	1.00	0.20	1.71	0.00

Sites: 1 (Austria, Danube River), 2 (Austria, Enns River), 3 (Germany, Inn River), 4 (Greece, Aliakmonas River), 5 (Greece, Aoös River), 6 (Greece, Sarantaporos River), 7 (Italy, Adige River), 8 (Romania, Cerna River), 9 (Romania, Mureş River), 10 (Slovakia, Danube River), 11 (Slovakia, Belá River).

^1^ Includes larvae infected with unidentified mermithid nematodes, as follows: 1 female (Site 1), 2 females and 1 male (Site 3, April), 1 female and 4 males (Site 3, October), 5 females and 2 males (Site 5), 3 females and 3 males (Site 7), and 3 females (Site 9).

^2^ Includes 1 female larva from 11 May 2011 and 2 female larvae from 9 May 2015, combined.

^3^ IS-1 was autosomal only at Site 4; at all other sites it was absent or linked to the X chromosome and, therefore, its frequencies are not shown in this table for those sites.

^4^ IS-1 and IS-4 were completely linked and in Hardy-Weinberg equilibrium (χ^2^ = 0.69, P > 0.05, df = 1): IS-1, 4 (ss = 10, si = 19, ii = 5, where s = standard and i = inverted).

^5^ Hardy-Weinberg equilibrium (P > 0.05, df = 1): IIL-14 (April: ss = 3, si = 13, ii = 10, χ^2^ = 0.16; October: ss = 1, si = 13, ii = 12, χ^2^ = 1.24), IIIL-4 (April: ss = 1, si = 11, ii = 14, χ^2^ = 0.43; October: ss = 7, si = 12, ii = 7, χ^2^ = 0.15).

^6^ IIIL-4 was linked to the Y chromosome in cytoform ‘C’ and, therefore, its frequencies are not shown in this table.

**Table 3:** Number of larvae of *Simulium colombaschense* for each sex-chromosome combination.

	Site 1	Site 2	Site 3 Apr	Site 3 Oct	Site 4	Site 5	Site 6	Site 7	Site 8^1^	Site 9	Site 10	Site 11
Cytoform	A	A	A	A	C	D	D	B	A	A	A	E
X_0_X_0_^2^					23^3^	13	1					
X_0_X_2_				1								
X_0_X_3_			1									
X_0_X_5_				1								
X_0_X_7_						2						
X_0_X_8_						1						
X_0_X_9_						1						
X_0_X_10_						2	1					
X_0_X_11_						2	1					
X_1_X_1_												1^4^
X_1_X_2_		1	3								1	
X_1_X_3_			1	2								
X_1_X_6_									2^4^	2^4^		
X_2_X_2_			4	6				4			3	
X_2_X_3_	1		4	2							1	
X_2_X_5_				1				1				
X_4_X_7_							1					
X_6_X_6_									1^4^	3^4^		
X_0_Y_0_					4	1						
X_0_Y_3_						11	4					
X_0_Y_4_					3							
X_0_Y_5_					4							
X_1_Y_1_	2	1	2									
X_2_Y_0_								6				
X_2_Y_1_	1		10	11							2	
X_3_Y_1_			1	2								
X_7_Y_0_						1						
X_7_Y_2_							1					
X_11_Y_0_						1						

Sites: 1, 2 (Austria), 2 (Germany), 4, 5, 6 (Greece), 7 (Italy), 8 (Romania, Cerna River), 9 (Romania, Mureş River), 10 (Slovakia, Danube River), 11 (Slovakia, Belá River).

^1^ Includes 1 female larva from 11 May 2011 and 2 female larvae from 9 May 2015, combined.

^2^ X_0_ = no X-linked rearrangement

X_1_ = IS-1

X_2_ = IS-1, 3

X_3_ = IS-1, 4

X_4_ = IS-5

X_5_ = IS-1, 3, 6

X_6_ = IS-1, 4, 8

X_7_ = IS-9

X_8_ = IS-10

X_9_ = IS-15

X_10_ = IS-16

X_11_ = IS-9, 16

Y_0_ = no Y-linked rearrangement

Y_1_ = IS-7

Y_2_ = IS-12, 13, 14

Y_3_ = IS-11, 12, 13, 14

Y_4_ = IIIL-4

Y_5_ = IIIL-4, 13

^3^ One female was a sex-exceptional IIIL-4 heterozygote.

^4^ Males are required to test the hypothesis of sex linkage of the specified inversions.

### Fixed Sequences

The banding pattern of *S*. *colombaschense* differed from the *Simulium* subgeneric standard by 12 fixed inversions: *IS-2*, *IL-1*, *IL-2*, *IL-6*, *IL-7*, *IIS-1*, *IIL-1*, *IIL-2*, *IIIS-1*, *IIIL-2*, *IIIL-3*, and *IIIL-5*. We regard *IIIS-1* as essentially fixed although it was absent in 1 homologue of a female larva from Slovakia (Site 9).

**Fig 2:**
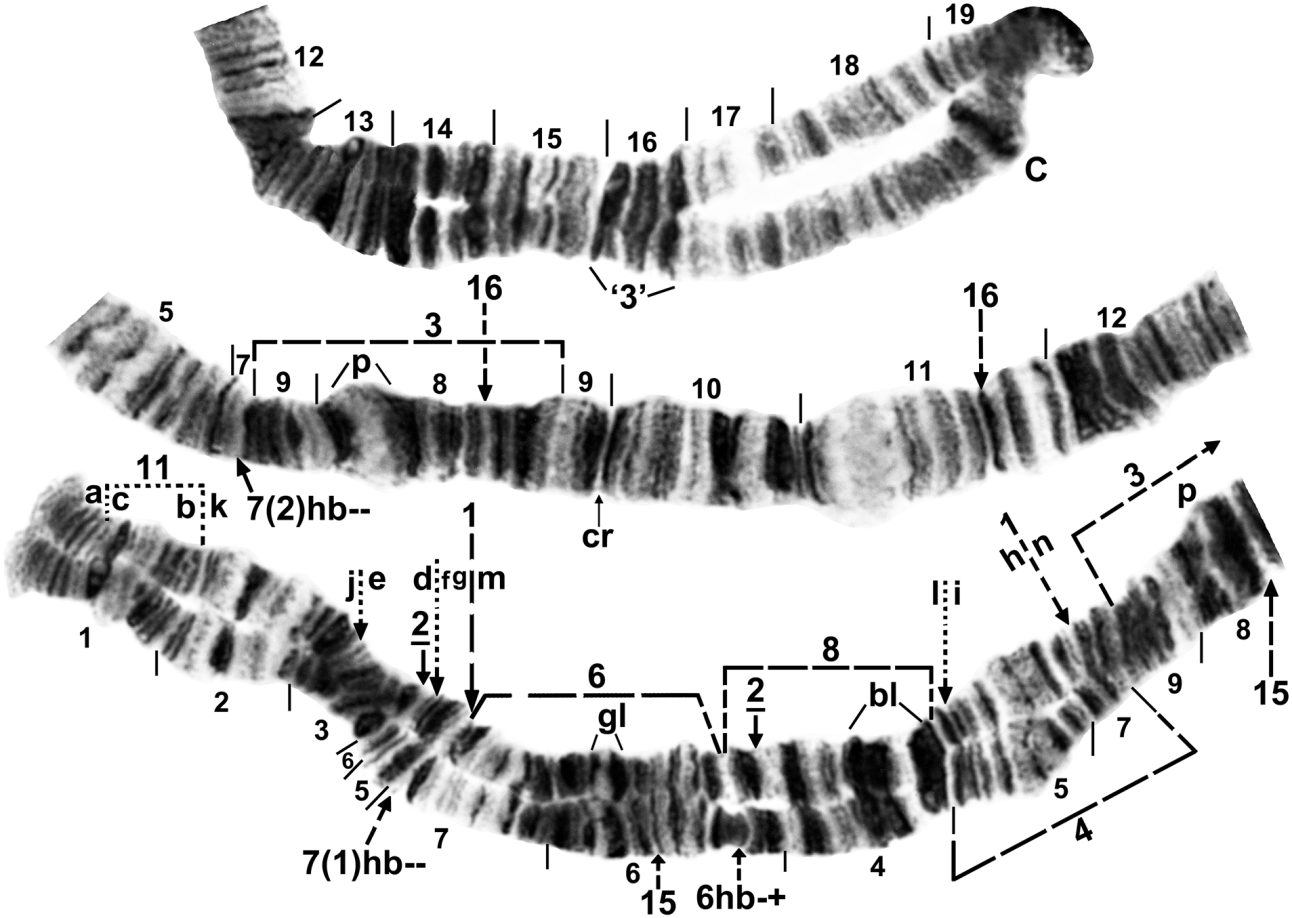
Chromosome arm IS of *Simulium colombaschense*, showing the common X sequence (X_2_X_2_) of cytoform ‘A’. Relative to the *Simulium* subgeneric standard [24], fixed inversion *IS-2* and X-linked IS-1, IS-3, and heteroband 6hb (heterozygous) are present. Breakpoints of X-linked inversions IS-4, IS-6, IS-8, IS-15, and IS-16 are indicated by dashed brackets or arrows, and the locations of X-linked heterobands 7(1)hb and 7(2)hb are shown by dashed arrows. Although IS-1 is depicted as X linked, it is autosomally polymorphic in cytoform ‘C’. Breakpoints of the 4 Y-linked inversions in cytoform ‘D’—IS-11, 12, 13, 14—are shown with dotted arrows. The predominant Y sequence (Y_3_) of cytoform ‘D’ can be derived from the IS-1, *IS-2* sequence by alphabetically ordering fragments ‘a’ through ‘n’. The actual Y_3_ sequence occurs on top of *IS-2*, but IS-1 is absent from the Y_3_ sequence; IS-1 is identified here by the g|m and h|n breakpoints. The Y_3_ sequence also includes an intercalated band (not shown) at the k|l junction; bl = 2 blocks, C = centromere, cr = crack, gl = glazed band, p = puffing band, ‘3’ = 3 marker.

**Fig 3:**
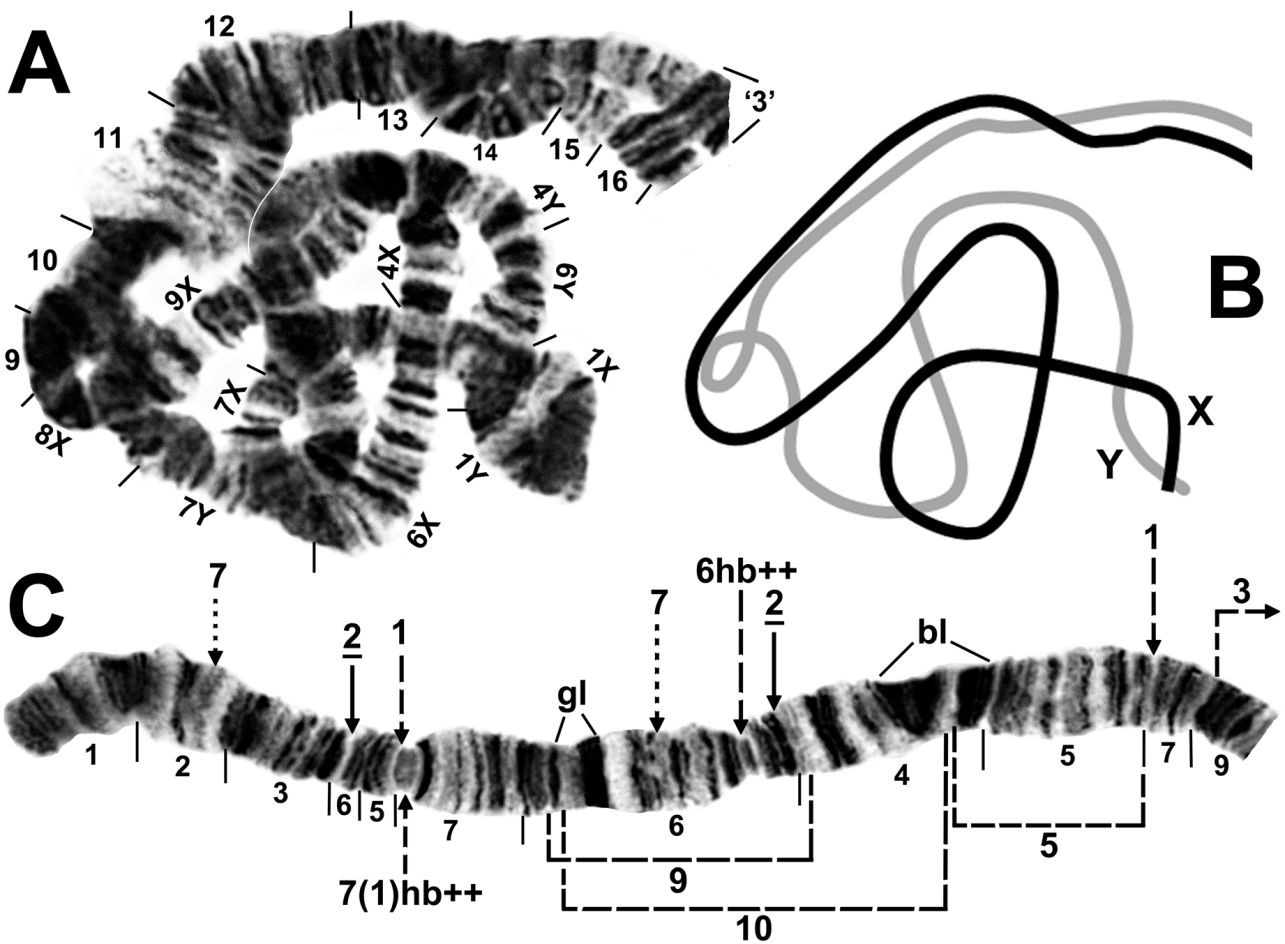
Chromosome arm IS, the sex arm, of *Simulium colombaschense* cytoform ‘A’. A. Typical X_2_Y_1_ inversion complex in cytoform ‘A’, formed by the X_2_ (IS-1, 3) chromosome coupled with the Y_1_ (IS-7) chromosome. Section numbers in the heterozygous region are followed by ‘X’ or ‘Y’ to indicate the sections of the X and Y chromosomes, respectively; ‘3’ = 3 marker. B. Interpretive idiogram of Fig 3A, showing configuration of the X_2_ (black) and Y_1_ (gray) homologues of a male larva. C. The common X_2_ (IS-1, 3) sequence of cytoform ‘A’. Relative to the *Simulium* subgeneric standard [24], fixed inversion *IS-2*, X-linked IS-1 and beginning of IS-3, and heterobands 6hb (homozygous) and 7(1)hb (homozygous) are present. X-linked IS-5, IS-9, and IS-10 are indicated by dashed brackets. Breakpoints of the Y-linked IS-7 inversion are shown by dotted arrows. Although the breakpoints are shown on the IS-1 X sequence, IS-7 occurs without IS-1; bl = 2 blocks, gl = glazed band.

**Fig 4:**
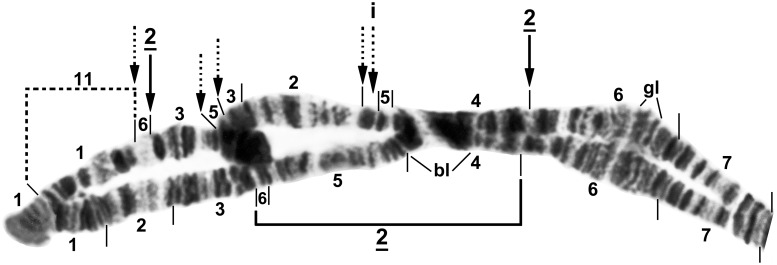
Chromosome arm IS, the sex arm, of *Simulium colombaschense* cytoform ‘D’. Male larva from the Aoös River, Greece. The undifferentiated X (X_0_, lower homologue) coupled with Y_3_ (IS-11, 12, 13, 14; upper homologue) on top of fixed inversion *IS-2*. Most of section 5 on the Y is obscured in a small loop. Dotted bracket and arrows mark the breakpoints of Y-linked inversions, and solid arrows and bracket mark the breakpoints of *IS-2*; bl = 2 blocks, gl = glazed band, i = Y-linked intercalated band.

**Fig 5:**
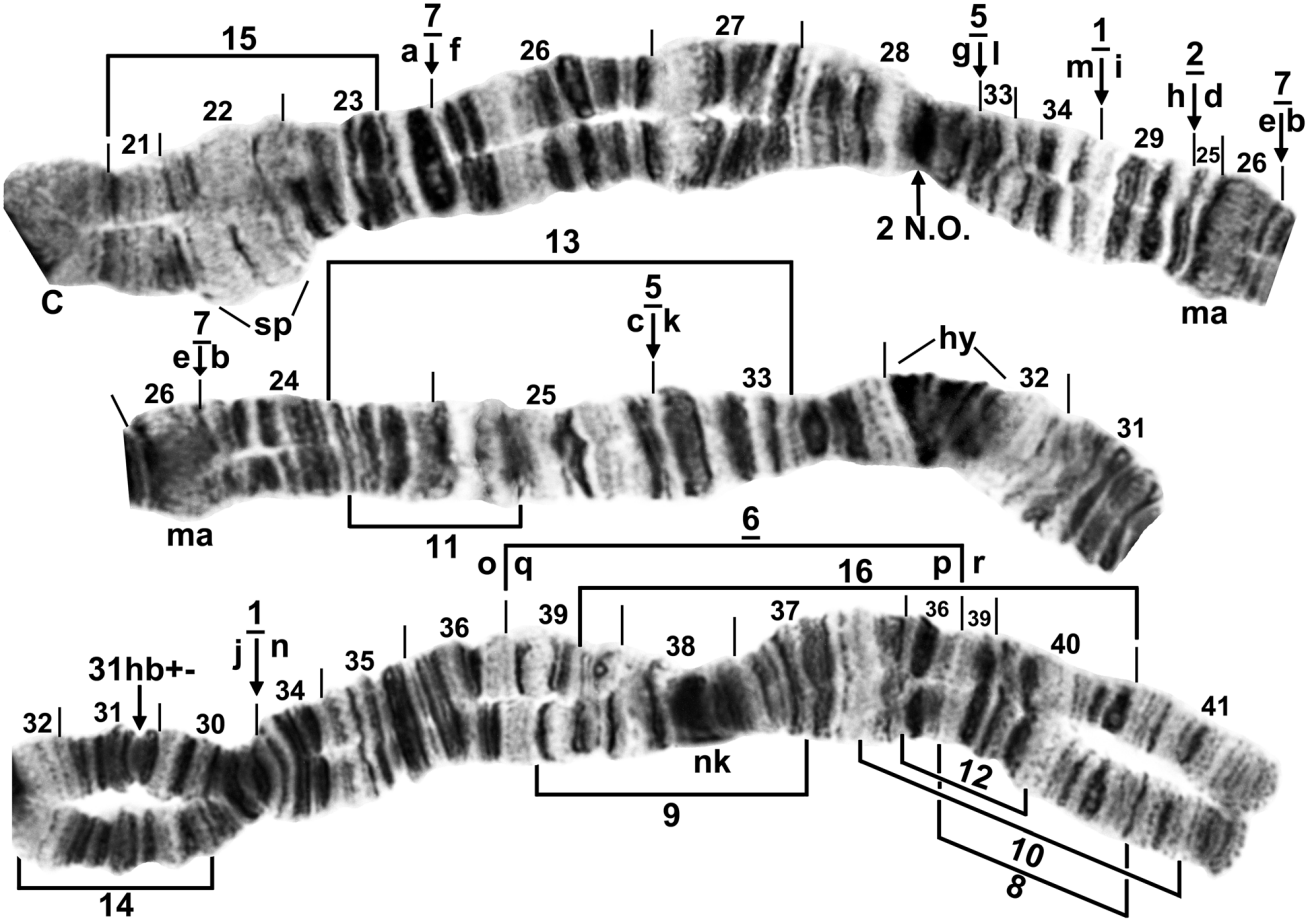
Chromosome arm IL of *Simulium colombaschense*. Upper and middle images: male larva, lower image: female larva. Relative to the *Simulium* subgeneric standard [24], fixed inversions *IL-1*, *IL-2*, *IL-6*, and *IL-7*, autosomal inversion IL-5 (shown here as fixed [underlined] in cytoform ‘A’, but polymorphic or absent in other cytoforms), and heteroband 31hb (heterozygous) are present. *IL-2* has only one breakpoint (arrow) shown on the map; the second breakpoint is obscured by IL-5, but would appear at the c|g junction in the absence of IL-5. The *Simulium* subgeneric standard sequence can be obtained from the *IL-1*, *IL-2*, *IL-6*, *IL-7*, IL-5 sequence by alphabetically ordering fragments indicated by small letters ‘a’ through ‘r’. Autosomal inversions IL-8 through IL-16 are indicated by brackets, and the position of a secondary nucleolar organizer (2 N.O.) is indicated by an arrow. C = centromere, hy = heavy, ma = marker, nk = neck, sp = spongy.

Fixed inversions in IS of *S*. *colombaschense* were nonoverlapping. *IS-2* typically was overlapped by one or more sex-linked inversions (Figs 2–4), but by itself moved the ‘2 blocks’ marker slightly more proximal to the centromere. IL was removed from the standard by 4 fixed inversions, although by 5 inversions in the Danube Basin and Italy’s Adige River where IL-5 was fixed or nearly so. These 5 inversions divided the arm into 10 fragments (Fig 5). The most parsimonious reassemblage of fragments to produce the subgeneric standard, one inversion step at a time, is shown below, where the order of fragments is represented by the letters ‘a’ through ‘r,’ corresponding with the lettering in Fig 5, and where brackets represent the inversion in each sequence. The order of inversions is constrained by *IL-1* and *IL-*2, which also are found in all or some studied relatives, respectively, and, therefore, must have been the first inversions to have occurred in IL during the evolutionary derivation of *S*. *colombaschense* from an ancestor, and by IL-5, which because it is not fixed in all populations, must have been the last inversion in the series.

Inversion 5 (*Simulium colombaschense* cytoforms ‘A’, ‘B’): a / fg **[**lm / ih / de / bc**]** kj / no / qp / r

Inversion *6* (*Simulium colombaschense* cytoforms ‘C’, ‘D’, ‘E’): a / fg / cb / ed / hi / mlkj / no **[**qp**]** r

Inversion *7*: a **[**fg / cb**]** ed / hi / mlkj / nopqr

Inversion *2*: abc **[**gfed**]** hi / mlkj / nopqr

Inversion *1*: abcdefghi **[**mlkj**]** nopqr

*Simulium* subgeneric standard: abcdefghijklmnopqr

**Fig 6:**
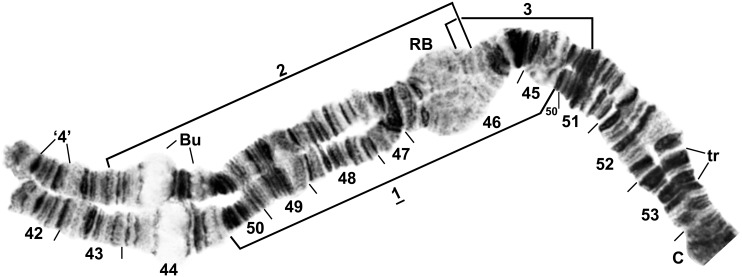
Chromosome arm IIS of *Simulium colombaschense*. Sections 42–49 and 52–53: female larva; remainder: male larva. Relative to the *Simulium* subgeneric standard [25], fixed inversion *IIS-1* is present. Autosomal inversions IIS-2 and IIS-3 are indicated by brackets. Bu = bulges, C = centromere, RB = ring of Balbiani, tr = trapezoidal, ‘4’ = 4 doublets.

**Fig 7:**
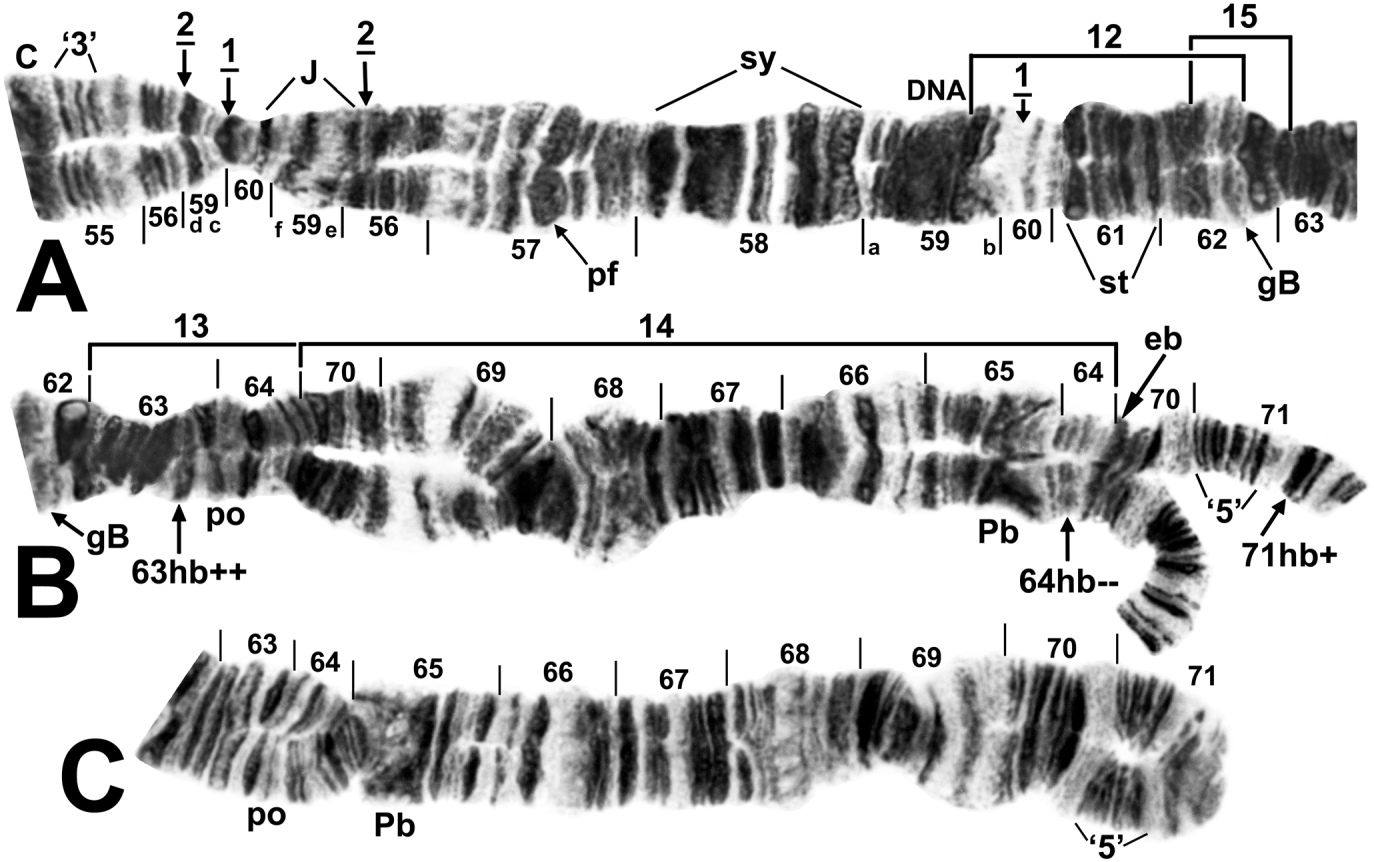
Chromosome arm IIL of female larva of *Simulium colombaschense*. A, B. Relative to the *Simulium* subgeneric standard [24], overlapping fixed inversions *IIL-1* and *IIL-2*, autosomal inversion IIL-14, heterobands 63hb (homozygous) and 71hb (heterozygous), and expressed band eb (homozygous) are present. Autosomal inversions IIL-12, IIL-13, and IIL-15 are indicated by brackets and the location of heteroband 64hb by an arrow. The 3 fragments of section 59 can be reassembled into the *Simulium* subgeneric standard sequence by alphabetically ordering the small letters ‘a’ through ‘f.’ C. Distal half of arm, showing the standard sequence (male larva from Sarantáporos River, Greece). C = centromere, DNA = DNA puff, gB = gray band, Pb = parabalbiani, pf = puffing band, po = polar, st = sawtooth, sy = symmetrical, ‘3’ = 3 sharp, ‘5’ = 5 sharp.

**Fig 8:**
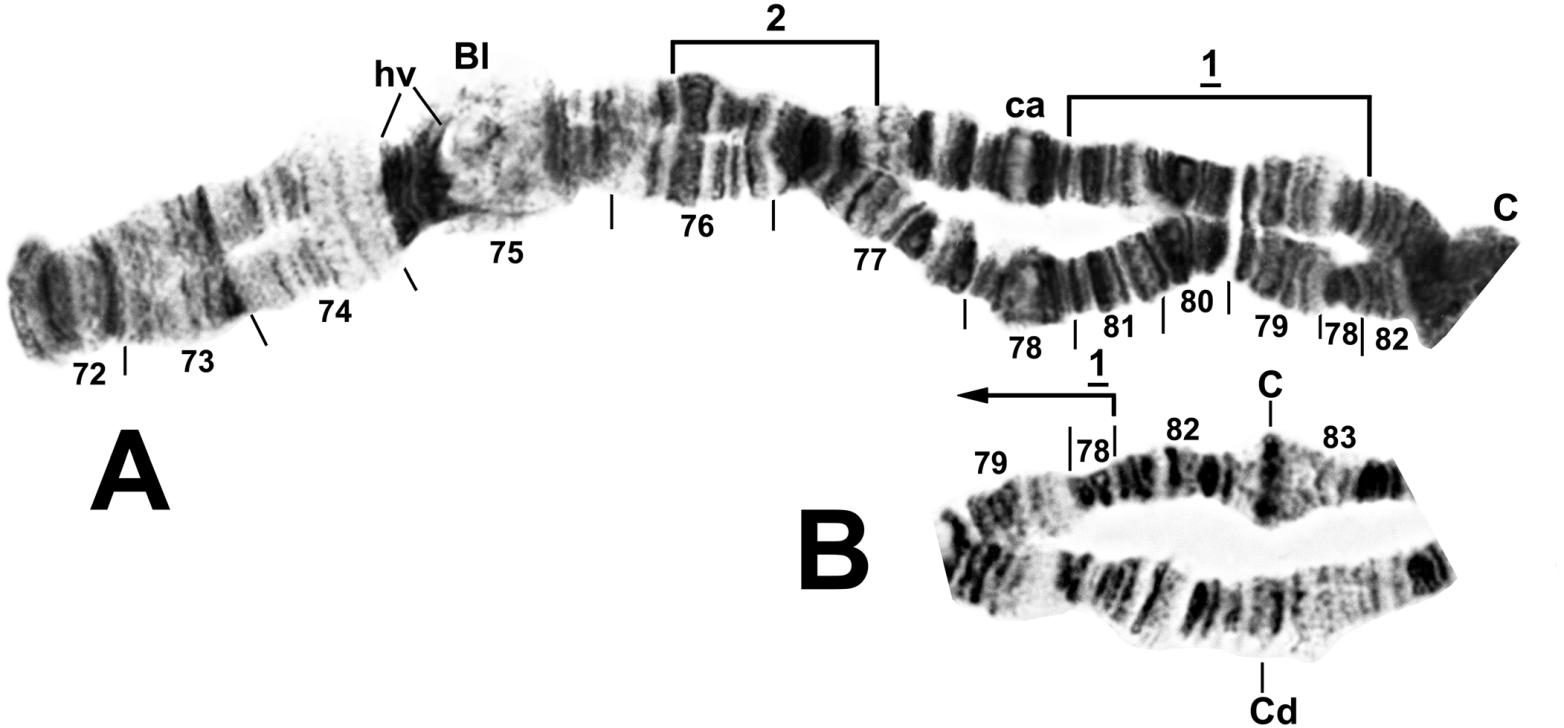
Chromosome arm IIIS of female larva of *Simulium colombaschense*. A. Relative to the *Simulium* subgeneric standard [24], *IIIS-1* is present. Autosomal inversion IIIS-2 is indicated by a bracket. B. Centromere region showing heterozygous arrangement of the centromere dimorphism and proximal breakpoint of *IIIS-1*. Bl = blister, C = centromere, ca = capsule, C_d_ = diffuse centromere band, hv = heavy group.

**Fig 9:**
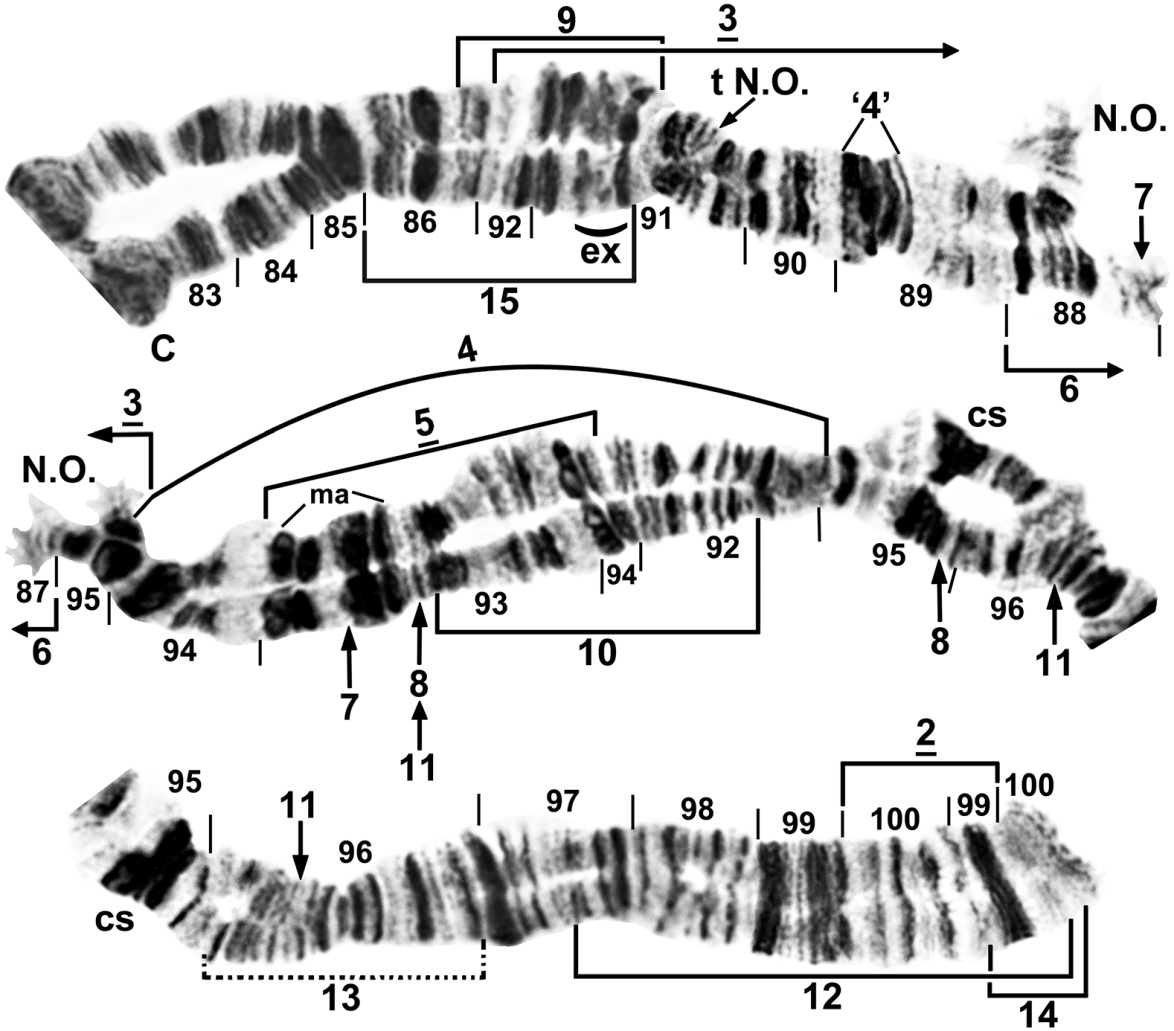
Chromosome arm IIIL of *Simulium colombaschense*. Middle image and sections 99–100: male larva; remainder: female larva. Relative to the *Simulium* subgeneric standard [25, 26], fixed inversions *IIIL-2*, *IIIL-3*, and *IIIL-5*, and autosomal (or Y-linked in ‘C’) inversion IIIL-4 are present. Autosomal inversions IIIL-6, IIIL-9, IIIL-10, IIIL-12, IIIL-14, and IIIL-15 are indicated by brackets, and breakpoints of IIIL-7, IIIL-8, and IIIL-11 by arrows; IIIL-7, IIIL-8, IIIL-10, and IIIL-11 do not occur on IIIL-4. Y-linked inversion IIIL-13 in cytoform ‘C’ is indicated with a dotted bracket. Position of a transposed nucleolar organizer (t N.O.) is indicated by an arrow. C = centromere, cs = cup and saucer, ex = exploded, ma = marker, N.O. = nucleolar organizer, ‘4’ = 4 bands.

The single fixed inversion in the IIS arm, *IIS-1*, occupied approximately half of the arm’s length (Fig 6). IIL differed from the standard sequence by two overlapping basal inversions, *IIL-1* and *IIL-2* (Fig 7). IIIS carried fixed inversion *IIIS-1* (Fig 8), and IIIL had 3 nonoverlapping inversions (Fig 9).

**Fig 10:**
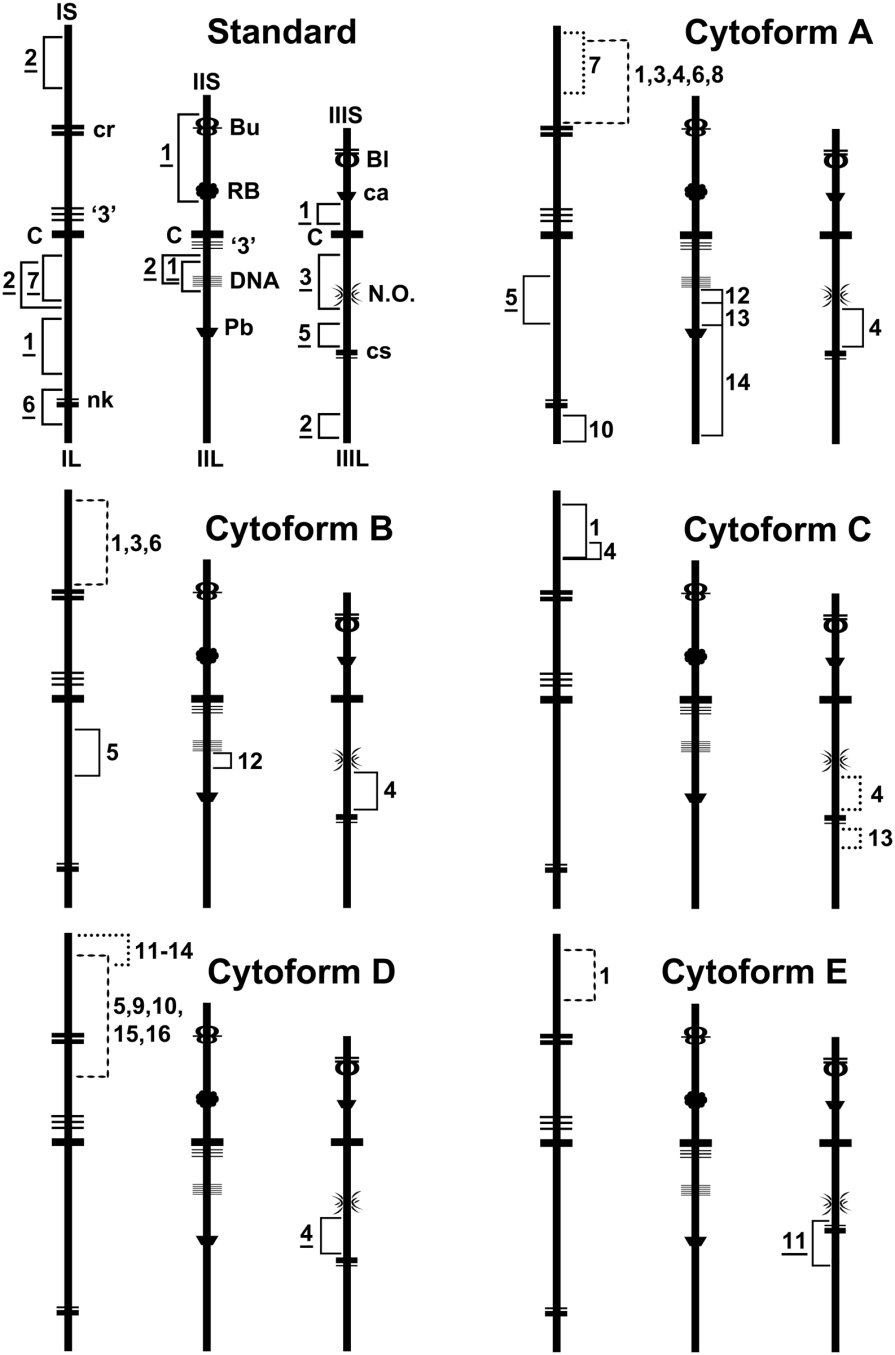
Idiograms of *Simulium colombaschense* and its cytoforms, showing diagnostic chromosomal features. ‘Standard’ represents the derivation of *S*. *colombaschense* from the standard sequence of the subgenus *Simulium* via 12 fixed inversions that are shared by all cytoforms. Idiograms of individual cytoforms show only diagnostic differences from this fundamental sequence, including all fixed inversions (underlined and bracketed on left side of chromosomes), all sex-linked inversions (X and Y linkage indicated by dashed and dotted brackets, respectively), and autosomal polymorphisms (bracketed on right side of chromosomes) with frequencies greater than 0.20. Heterobands are not shown. Selected landmarks are indicated as follows: Bl = blister, Bu = bulge, C = centromere, ca = capsule, cr = crack, DNA = DNA puff, nk = neck, Pb = parabalbiani, RB = ring of Balbiani, ‘3’ (on IS) = 3 marker, ‘3’ (on IIL) = 3 sharp.

The 12 fixed inversions, relative to the *Simulium* subgeneric standard, provided the fundamental sequence for recognizing additional diversity within *S*. *colombaschense* (Fig 10). We recognize 5 cytoforms (‘A’, ‘B’, ‘C’, ‘D’, and ‘E’) among the 11 studied populations. Each cytoform was defined based primarily on the sex chromosomes, supported by autosomal features and a limited number of fixed inversions.

### Cytoform ‘A’

Populations in the Danube River and its tributaries (Sites 1–3, 8), including the Mureş River (Site 9), which joins the Danube via the Tisa River, all within about 75–500 km of one another, were chromosomally cohesive across space and time (April–October). Cytoform ‘A’ was characterized by fixation of IL-5, an X chromosome typically based on IS-1, and a Y that carried IS-7. Although males were lacking in our samples from Romania’s Cerna and Mureş Rivers, we assigned larvae from these sites to cytoform ‘A’, based on fixation of IL-5, a probable X chromosome based on IS-1, and the presence of autosomal polymorphisms IIL-13 and IIIC_d_, which were shared with populations in the Danube River.

#### Autosomal polymorphisms

Cytoform ‘A’ had 17 autosomal floating inversions and the highest levels of autosomal heterozygosity among the 5 cytoforms (Table 2). Inversions IIL-14 and IIIL-4 were in Hardy-Weinberg equilibrium for the two seasonal samples from Germany (Table 2). Three inversions varied clinally from the upper Danube to the Iron Gate: IIL-13 increased from a frequency of 0.02–0.04 in the upper Danube to 0.94 in Romania, whereas IIL-14 and IIIL-4 decreased from 0.50 and higher to 0.00. IIL-14 was associated with a band expression (eb) at the junction of sections 64 and 70 (Fig 7); whether the band was an enhancement (hb) of a fine band or a novel, intercalated band (i) could not be determined. Autosomal heterobands included the pale-staining 31hb in all samples (Fig 5), with frequencies as high as 0.57 per site. Heteroband 71hb in IIL (Fig 7) and a telomeric heteroband in IIIL occurred heterozygously in a female (28 April) and male (24 October), respectively, from Germany (Site 3). The centromere band of chromosome III was heterozygously diffuse (IIIC_d_, Fig 8) in German larvae (2 females, 1 male) on both sampling dates and in 1 female larva from Romania’s Cerna River (Site 8). A male larva from Slovakia (Site 9) had a secondary nucleolar organizer in section 28 (Fig 5), and 1 female larva from the same site had the primary nucleolar organizer transposed, without an inversion, to section 91 on the IIIL-4 homologue (Fig 9).

#### Sex chromosomes

The 7 sex chromosomes (X_0_, X_1_, X_2_, X_3_, X_5_, X_6_, and Y_1_) of cytoform ‘A’ were represented in 13 zygotic combinations (Table 3, Figs 2 and 3). Of 114 X sequences, 2.6% had no sex-linked inversions (X_0_), and 14% carried only IS-1 (= X_1_). The remaining X sequences had at least one additional inversion grafted onto IS-1. Of these, X_2_ (IS-1, 3) was the most common with 57.0% representation, followed by X_3_ (IS-1, 4) with 13.2%, X_6_ (IS-1, 4, 8) with 11.4%, and X_5_ (IS-1, 3, 6) with 1.8%. The 3 most common X sequences (X_1_, X_2_, and X_3_) frequently had one or two heterobands; 58.3% of these X sequences had heteroband 6hb and 36.5% had 7(1)hb. Both bands showed variable expression, from imperceptibly thin to thick; 6hb stained darkly, whereas 7(1)hb was typically pale and glassy (Figs 2 and 3). The heterobands were not found on X_4_, X_5_, X_6_, the Y chromosome, or in 7 of 8 Romanian larvae from Sites 8 and 9 (1 larva from Site 8 had 6hb). The monomorphic Y chromosome (Y_1_) was consistently linked with IS-7 and lacked heterobands.

### Cytoform ‘B’

This cytoform, from the Adige River in Italy (Site 7), was similar to cytoform ‘A’ in its basic sequence and X chromosome, which minimally carried IS-1, 3. It was characterized by a Y chromosome with no sex-linked inversions (Table 3, Fig 2).

#### Autosomal polymorphisms

IL-5 was nearly fixed (frequency = 0.95). Of the 4 additional autosomal inversions, 3 were shared with cytoform ‘A’ (Table 2). Heterobands 31hb and 64hb each occurred (in different larvae) with a frequency of 0.09. The centromere band of chromosome III was heterozygously diffuse (C_d_, Fig 8) in 1 male larva; this polymorphism was shared with cytoform ‘A’.

#### Sex chromosomes

The sex chromosomes X_2_ (IS-1, 3), X_5_, (IS-1, 3, 6), and Y_0_ were found in 3 combinations (Table 3). Heterobands 6hb and 7(1)hb were absent in males and in high frequency in females: 100% and 76.9%, respectively. A glassy heteroband band, 7(2)hb (Fig 2), was found in one female larva.

### Cytoform ‘C’

Site 4 on the Aliakmonas River in Greece was pure for cytoform ‘C,’ which was characterized by a high frequency (0.43) of the linked autosomal inversions IS-1 and IS-4 and sex determination based on IIIL. The only triploid larva in our study, a female of ‘C’, was heterozygous for IS-1, 4, with only 1 of the 3 homologues carrying the inverted sequence.

#### Autosomal polymorphisms

Three autosomal inversions were found, of which the two most common (IS-1 and IS-4) were linked to one another (Table 2). Heterobands were absent in IS, but 63hb occurred in IIL of 3 larvae (Fig 7).

#### Sex chromosomes

This cytoform was the only one with sex determination based on IIIL (Fig 9) rather than IS (Table 3). The Y chromosome occurred in 1 of 3 forms: undifferentiated (Y_0_), with IIIL-4 (Y_4_), or with IIIL-4, 13 (Y_5_). The X chromosome (X_0_) carried no sex-linked rearrangements, although 1 sex-exceptional female, heterozygous for IIIL-4, was found.

### Cytoform ‘D’

This segregate, from Greece’s Aoös and Sarantaporos Rivers (Sites 5 and 6), was characterized by absence of IS-1, fixation of IIIL-4 (Table 2), unique Y chromosomes (Y_2_ and Y_3_), and 6 novel X chromosomes (Table 3, Figs 2 and 3C).

#### Autosomal polymorphisms

Cytoform ‘D’ had the fewest autosomal polymorphisms (4) and least heterozygosity (Table 2).

#### Sex chromosomes

The typical Y chromosome (Y_3_) of cytoform ‘D’ had 4 linked inversions: a complex of 3 overlapping inversions (IS-12, 13, 14) atop *IS-2* and the subterminal IS-11 (Fig 4). One larva lacked these inversions; its Y, therefore, was identical to X_0_. A second larva lacked IS-11, but had IS-12, 13, 14 (Y_2_). Several derivations of the 3 overlapping inversions from the *IS-2* sequence are possible; the 3 inversions, therefore, are not individually identified on our maps. Instead, precise breakpoints of all 4 Y-linked inversions (IS-11, 12, 13, 14) are shown in Figs 2 and 4. The breakpoints in Fig 2, however, are identified on the X-linked IS-1 sequence (although IS-1 is not present in cytoform ‘D’) and, therefore, the derivation of IS-11, 12, 13, 14 in Fig 2 is from a IS-1 condition, with IS-1 identified on our map by the g|m and h|n breakpoints. The complex Y paired most often (79% of males) with a simple X chromosome (X_0_; i.e., with no inversions on the fixed *IS-2* sequence). Six additional X chromosomes were found (Figs 2 and 3c): X_4_ (IS-5), X_7_ (IS-9), X_8_ (IS-10), X_9_ (IS-15), X_10_ (IS-16), and X_11_ (IS-9, 16). No heterobands were expressed on the X, but Y_2_ and Y_3_ each consistently had an extra band (i) (Fig 4) intercalated at the k|l junction (Fig 2).

### Cytoform ‘E’

A single female larva, tentatively recognized as a distinct cytoform, ‘E’, was collected from the Belá River of Slovakia (Site 11). It was homozygous for IS-1 (tentatively considered X linked), heteroband 31hb, and the unique IIIL-11 inversion (Table 2, Fig 9). It lacked IL-5 and heterobands in IS, setting it apart from cytoforms ‘A’ and ‘B’.

### Evolutionary Relationships

**Fig 11:**
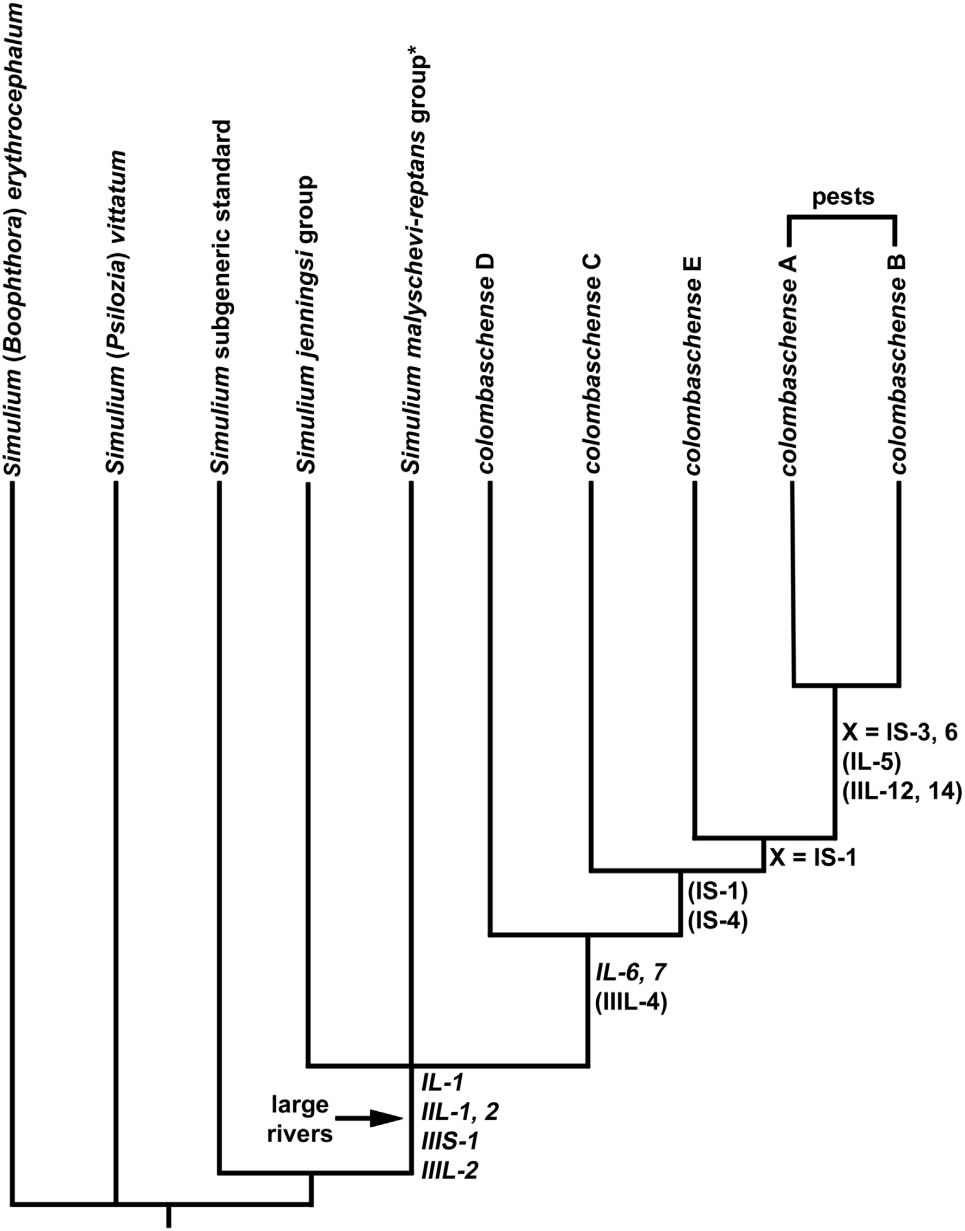
Cytophylogeny of the cytoforms of *Simulium colombaschense*. *The *Simulium malyschevi/reptans* group includes *S*. *colombaschense*. Only rearrangements that could be assessed in the outgroups are shown. Inversions in italics are fixed, those in parentheses are polymorphic, and those following “X =“ are sex linked.

One or both breakpoints of 11 inversions shared by 2 or more cytoforms of *S*. *colombaschense* could be evaluated in the outgroups: IS-1, *IS-2*, IS-3, IS-4, IS-6, IL-5, *IL-6*, *IL-7*, IIL-12, IIL-14, and IIIL-4 (Fig 11). Ten of these inversion sequences were absent in the outgroups. *IS-2*, however, was identical to or differed at most by one thin band from the sequence in *S*. *vittatum*; we, therefore conservatively treated *IS-*2 as the same sequence as in *S*. *vittatum*. The scrambled distal portion of IS of *S*. *erythrocephalum* did not allow us to evaluate the presence or absence of *IS-2*.

The 5 cytoforms of *S*. *colombaschense* formed a monophyletic group united by synapomorphic fixed inversions *IL-6* and *IL-7*. All cytoforms, except ‘D’, minimally shared IS-1, which was X linked in ‘A’, ‘B’, and possibly ‘E’. Cytoforms ‘A’ and ‘B’ were sister taxa, based on the presence of IL-5, IIL-12, and IIL-14, and by further differentiation of the X chromosome.

## Discussion

### Cryptic Taxa

Our uppermost collection of *S*. *colombaschense* in the Danube system is from the Inn River at the confluence with the Danube. From here downriver to the Balkan states, including the area of historical abundance at the Iron Gate, the Danube is colonized by *S*. *colombaschense* [32]. Our repeated efforts to find *S*. *colombaschense* at the Iron Gate have been unsuccessful. However, larvae of cytoform ‘A’ from Romania’s Cerna River, a Danube tributary within about 15 km of the Iron Gate, suggest that cytoform ‘A’ represents true *S*. *colombaschense* of historical notoriety. Similarly, the seven names synonymized with *S*. *colombaschense* [19] on the basis of morphological evidence, and applying to populations along the Danube, are supported by chromosomal evidence.

Cytoform ‘B,’ the Adige River population, might represent a distinct species characterized chiefly by its undifferentiated Y chromosome (Y_0_). However, the suite of chromosomal features shared with ‘A’—fixed inversions, X chromosomes, and common autosomal polymorphisms—supports a conservative view that ‘A’ and ‘B’ are a single species with geographic polymorphism of the Y chromosome. Sex-chromosome polymorphism is common within species of the Simuliidae [5, 29]. We, therefore, regard ‘B’ as a cytotype of ‘A’. Our sample of ‘B’ from the Adige River was taken about 380 km from our nearest collection site in the Danube Basin. We also have ethanol samples, not amenable for chromosomal analysis, from Fiume Rienza (46°48'55"N 11°44'12"E) in the Adige catchment and from the Isar River (47°30'22"N 11°17'16"E) in the Danube catchment, only 85 km apart, separated by the northern and central eastern Alps. However, high mountains rising to more than 3000 m between the Adige and Danube populations suggest the possibility of some degree of isolation of ‘B’.

Populations of cytoforms ‘C’ and ‘D’ in Greece, although as few as 50 km apart, are pure and distinct from one another. Among the 78 analyzed larvae, we found no evidence of interbreeding. IS-1 + IS-4 is common in ‘C’ but absent in ‘D’, IIIL-4 is Y linked in ‘C’ but fixed in ‘D’, and the sex chromosomes are nonhomologous (I versus III), a classic indicator of separate species [33]. Different species rarely share the same sex-chromosome system, although rearrangements that are sex linked in one species are frequently autosomal or fixed in another species [28]. Sex-chromosome differentiation has been postulated as a driver of speciation [30]. The Aoös and Aliakmonas drainages are separated by mountains that rise to 2400 m, although whether the mountains provide a barrier to dispersal is not known. Given the physical proximity of ‘C’ and ‘D’, without evidence of interbreeding, we suggest that they are full species, although their status vis à vis the other cytoforms is not known. Our collections of ‘C’ and ‘D’ are about 500 km south of the nearest sampling site for cytoform ‘A’. Neither of the two cytoforms in Greece is considered a pest. On the contrary, *S*. *colombaschense* had not been recorded in Greece before 2012 [34].

Establishment of a separate cytoform (‘E’), based on a single specimen from Slovakia’s Belá River, might seem unusual. The specimen, however, is chromosomally distinct and geographically remote (ca. 200 km) from the nearest Danube population. The Belá River is a tributary of the Váh River, which flows into the Danube. The Váh River at one time might have been suitable for colonization by *S*. *colombaschense*, but its riverbed and discharge patterns have been modified by a series of hydropower plants, and before 1990 the Váh was heavily polluted, especially by a pulp mill in its upper reaches. Notwithstanding the possible historical connection of populations of *S*. *colombaschense* in the Danube and the Belá, the recognition of cytoform ‘E’ carries the prediction that IL-5 is absent and IIIL-11 is fixed. The situation is reminiscent of cytoform ‘CKL’ of the Nearctic *S*. *tuberosum* complex, which initially was recognized as a possible sibling species on the basis of two larvae [35], and later was shown to be a valid species, *S*. *perissum* Dyar & Shannon, when more material came to hand [4, 36].

The taxonomic status of two putative species structurally similar to *S*. *colombaschense*—*S*. *liriense* Rivosecchi and *S*. *voilense* Sherban—is relevant to our investigation. *Simulium liriense* was described from the Liri River of Italy [37] about 480 km south of our sampling site in the Adige River and about 660 km across the Adriatic Sea from our collection site in the Aoös River. Thus, the name might be associated with cytoform ‘B’, ‘C’, or ‘D’, or with a chromosomally distinct entity. The species has not been collected since 1967 from either of the two rivers, the Liri or the Garigliano, where its immature stages originally were found [14], and our two attempts to collect it were futile. The Sacco River, Italy’s most polluted river, flows into the Liri, further degrading the habitat and setting up the possibility that *S*. *liriense* is extinct [38]; however, additional prospecting is warranted before making a final pronouncement on its status.

*Simulium voilense* was described from the Doftana River in Romania [39]. The primary diagnostic character used by most authors, in relation to *S*. *colombaschense*, is the pupal gill of 10, rather than 10–16, filaments [40]. In a June 1995 sample that we collected from the Danube below the mouth of the Inn River, 57% of 97 pupae had 10 filaments per gill, 8% had 11, 30% had 12, and 5% had 13. This filament distribution is similar to that in the Austrian Danube in April 2011 [40]. Our chromosomal sample of 28 April 2011 from the Inn River (cytoform ‘A’) had 3 larvae with dark gill histoblasts of which 1 larva had 11 filaments and 2 larvae had 12 filaments; the 24 October sample had 6 larvae with dark gill histoblasts of which 4 had 10 filaments and 2 had 12 filaments. We, therefore, suspect that reports of *S*. *voilense* in Slovakia [41] and Italy [42, 43], which are based solely on the number of gill filaments, refer to true *S*. *colombaschense*. An electrophoretic analysis could not differentiate Italian pupae with 10 filaments from those with 12–14 filaments [44]. The variable filament number in chromosomally cohesive populations demonstrates that the number of gill filaments is not a diagnostic character for *S*. *voilense* even though in our material (*n* = 104), specimens with 10 filaments (58.7%) and 12 filaments (32.7%) represent two clusters, with only 3.8% having 11 filaments and 4.8% having 13 filaments. An even number of gill filaments is disproportionately represented across the Simuliidae [4]. The validity of *S*. *voilense* as a distinct species remains in question, and will require comparative material from its type locality in Romania.

### Evolutionary Relationships

*Simulium colombaschense* is a member of the *S*. *jenningsi*-*malyschevi*-*reptans* clade. This lineage is uniquely defined by inversions *IIL-1*, *IIL-2*, *IIIS-1*, *IIIL-2*, and perhaps also *IL-1* and *IIS-2* [21]. *IL-2* is shared minimally with *S*. *acrotrichum* Rubtsov [21] and the *S*. *reptans* complex (unpublished). The occurrence of one female Slovakian larva heterozygous for *IIIS-1* is anomalous; the inversion is fixed in all previously studied members of the *S*. *jenningsi-malyschevi-reptans* clade. Either the Slovakian larva carried a mimic inversion of *IIIS-1*, with imperceptibly different breakpoints, or the standard sequence is a rare, ancestral polymorphism in some clade members.

*IS-2*, although unique among chromosomally examined species of the subgenus *Simulium*, is similar, if not equivalent, to the standard sequence in *S*. *vittatum*. The *IS-2* sequence, or its mimic, is also present in Neotropical taxa, such as the *S*. (*Psilopelmia*) *perflavum* group [45], *S*. (*Psaroniocompsa*) *quadrifidum* group [46], and *S*. (*Psaronicompsa*) *inaequale* group (unpublished). The absence of *IS-2* in all other examined members of the *S*. *jenningsi*-*malyschevi*-*reptans* clade, and more inclusively in all studied species of the subgenus *Simulium*, suggests that *IS-2* in *S*. *colombaschense* is a mimic inversion rather than uniquely shared with *S*. *vittatum* and other nonmembers of the subgenus *Simulium*.

The *IIIL-3* inversion, identified previously as having potential phylogenetic importance [21], is gaining additional traction as a defining synapomorphy for a monophyletic lineage within the *S*. *jenningsi-malyschevi-reptans* clade. *IIIL-3* is uniquely derived in a group of species, minimally including *S*. *acrotrichum*, the *S*. *arcticum* complex, *S*. *colombaschense*, *S*. *defoliarti* Stone & Peterson, *S*. *flavidum* Rubtsov, the *S*. *reptans* complex [21], and *S*. *murmanum* Enderlein (unpublished). *IIIL-3* is not present in *S*. *cholodkovskii* Rubtsov (unpublished), *S*. *decimatum* Dorogostaisky, Rubtsov & Vlasenko, *S*. *malyschevi* Dorogostaisky, Rubtsov & Vlasenko, or *S*. *subvariegatum* Rubtsov [21], which historically have been lumped with some members of the *IIIL-3* line. Thus, the *S*. *malyschevi* and *S*. *reptans* groups, as established morphologically [20] and as currently recognized [19, 47], are apparently ill defined and not independently monophyletic. Monophyly of the *S*. *jenningsi* group, however, is well supported [48].

*IIIL-5* is shared with the *S*. *reptans* complex (unpublished) and possibly also with *S*. *acrotrichum*; the breakpoints of *IIIL-5* are nearly identical to those at the o|g and p|j breaks within the IIIL inversion complex of *S*. *acrotrichum* (fig 8 of Adler & Huang [21]). *IIIL-5*, however, is lacking in the *S*. *arcticum* complex, *S*. *defoliarti*, and *S*. *murmanum* (unpublished). Thus, among chromosomally known species, the *S*. *reptans* complex and *S*. *acrotrichum* are in a trichotomy with *S*. *colombaschense*. *Simulium acrotrichum* and *S*. *colombaschense* are allopatric, but the *S*. *reptans* complex is sympatric with *S*. *colombaschense* over the entire range of the latter.

The IS-1 inversion provides a classic example of the different fates an inversion can take in the differentiation of evolutionary lines from an ancestral population [24, 26]. The inversion is X-linked in ‘A’, ‘B’, and possibly ‘E’; autosomally polymorphic in ‘C’; and absent (lost?) in ‘D’. IIIL-4 also appears in different roles: Y-linked in ‘C’; fixed in ‘D’; autosomally polymorphic in ‘A’ and ‘B’; and possibly absent in ‘E’.

### Paradox of Pests and Conservation

The macrogenome of *S*. *colombaschense* reveals an irony: The name *S*. *colombaschense*, long associated with history’s most notorious simuliid pest, includes two or more nonpest species (e.g., ‘C’ and ‘D’) that could be vulnerable to loss (extinction?) if their limited breeding habitats are corrupted. The reality of this possibility is demonstrated by the apparent loss of *S*. *liriense* from much, if not all, of its original habitat. If *S*. *liriense* is a unique species or cytoform and is now lost, it is one of a few examples [49] of a black fly driven to extinction by human agency. Even the superabundant *S*. *colombaschense* was eradicated from its original pest-producing habitat in the Iron Gate by habitat alteration, viz., impoundment [9].

### Origins of Pest Status

In the Simuliidae, pest status typically is reached by either of two pathways: (1) colonization of large continental rivers more than 100 m wide (specialization) or (2) colonization of a wide variety of smaller streams and rivers (generalization). The essential determinant of pest status in each case is the production of population levels sufficient to exceed an economic threshold. Either pathway can be enhanced by the construction of impoundments with epilimnetic release [3, 50, 51], which increases food quality and abundance [52].

Of the roughly 40 most significant simuliid pests of humans and domesticated animals [6], about one-third are breeding specialists of the world’s large rivers, including seven species in the *S*. *jenningsi*-*malyschevi*-*reptans* clade. At least 14 (18%) of the 77 nominal species in this clade colonize some of the world’s largest rivers, including the majority of species (*S*. *colombaschense*, *S*. *kurense* Rubtsov & Djafarov, *S*. *luggeri* Nicholson & Mickel, *S*. *reptans* (L.), and *S*. *vampirum* Adler, Currie & Wood) responsible for simuliotoxicosis [7, 20]. Among the five cytoforms of *S*. *colombaschense*, Cytoform ‘A,’ the pest of historical infamy, inhabits the largest rivers (e.g., Danube), whereas three of the cytoforms (e.g., ‘C’, ‘D’, and ‘E’) in the smaller rivers are not pests. Of the nine big-river species in the clade, which have not caused simuliotoxicosis, at least three (*S*. *cholodkovskii*, *S*. *jenningsi* Malloch, and *S*. *penobscotense* Snoddy & Bauer) are major, but nonlethal, annoyance pests of humans and domesticated animals [4, 20]. Notable among them is *S*. *jenningsi*, which colonizes the biggest rivers of eastern North America, and has been the target of one of the world’s largest pest-management programs for simuliids [4]. Evolutionary acquisition of the ability to colonize the world’s great rivers, such as the Danube, thus represents a key step toward pest status: larger rivers produce larger simuliid populations.

Another one-third of the most significant pests are habitat generalists, colonizing a majority of small streams and mid-sized rivers in an area. These pests include species such as *P*. *mixtum* Syme & Davies, *S*. *erythrocephalum* (De Geer), *S*. *ornatum* Meigen, *S*. *pertinax* Kollar, *S*. *venustum* Say, and *S*. *vittatum* Zetterstedt [4, 53, 54]. Some of these habitat generalists sporadically colonize the largest rivers in an area and also can become major pests via the big-river pathway [50, 55–57].

Available evidence suggests that simuliids evolved in small streams of mountainous terrain [5], the predominant breeding habitat of extant members of the entire family. Colonization of rivers wider than 10 m is characteristic of the majority of species in the *S*. *jenningsi*-*malyschevi*-*reptans* clade. Outgroup comparisons indicate that the ability to colonize rivers, including Earth’s largest, arose in an ancestor of the *S*. *jenningsi*-*malyschevi*-*reptans* clade. Additional, independent origins of large-river colonization appear infrequently and sporadically in the phylogeny of the Simuliidae, and include other pests of historical prominence, such as *Cnephia pecuarum* (Dyar & Shannon) of North America’s Mississippi River Valley [4], *S*. (*Byssodon*) *maculatum* (Meigen) of the Palearctic Region [20], and *S*. (*Psaroniocompsa*) *amazonicum* Goeldi and *S*. (*P*.) *oyapockense* Floch & Abonnenc of Brazil [53]. *Metacnephia lyra* (Lundström), a spectacularly abundant species in large, unregulated rivers of northern Fennoscandia [58], illustrates the absence of pest problems when feeding habits do not conflict with human interests; *M*. *lyra* feeds on wild birds [59]. The influence of *M*. *lyra* on the lotic ecosystem and the native wildlife of an area, however, is considerable [60]. The effects on riverine fauna and local wildlife by the historically important big-river pests must have been similarly profound, particularly before introduction of domesticated animals provided alternative hosts.

Big-river colonization is associated with a set of structural, developmental, and behavioral characters. Structural characters include short, robust labral fans and antennae [4, 61], and a boot-shaped cocoon that protects the gill filaments from swift currents and abrasive sediment. The larval bodies of nearly all members of the *S*. *malyschevi* and *S*. *reptans* groups gradually expand posteriorly, which permits close packing of individuals on substrates [4], further enhancing the ability to achieve large populations. Large-river species without gradually expanded larval bodies, such as *S*. *jenningsi* and *S*. *penobscotense*, exploit the increased attachment areas afforded by the three-dimensional architecture of beds aquatic plant, such as *Potamogeton* [62, 63]. Colonization of large rivers provides access to rapid delivery of abundant, small food particles that favor dense populations [64] and decrease development time [58], facilitating production of multiple generations. Multivoltinism, in turn, promotes pest status by building up population levels over a season. The pests of the *S*. *jenningsi*-*malyschevi*-*reptans* clade are typically multivoltine, except *S*. *vampirum*, which has one generation annually with three cohorts, giving the appearance of multivoltinism [65]. The ability to inhabit the swiftest areas of big rivers confers the additional benefit of protection from predators [58].

Behavioral adaptations for large-river colonization, such as those related to dispersal and oviposition, are insufficiently understood. Some evidence suggests that big-river species travel far (> 50 km) for a bloodmeal [3, 62, 66]. Dispersal distances of more than 300 km from the river channel have been reported for host-seeking females of *S*. *colombaschense* [9]. These claims, however, should be viewed cautiously; at the time, *S*. *colombaschense* was not known from areas of the Danube beyond the Iron Gate. If the dispersal distances are reliable, the situation is reminiscent of some savanna members of the African *S*. *damnosum* complex that disperse more than 500 km [67].

We suggest that site fidelity is related positively to habitat specialization and, therefore, should differ between large-river specialists and stream-habitat generalists. As the size of a watercourse increases, the prevalence of watercourses of the corresponding size decreases; thus, the largest rivers are numerically scarcest. Selection should favor females that locate appropriate sites for preimaginal development. Perhaps the greatest assurance of finding appropriate habitat would be a return to the natal habitat. The return of females to natal waterways to oviposit would concentrate and build populations in particular habitats, exacerbating pest problems. It also would promote site fidelity and ecological isolation, potentially leading to population differentiation, including speciation [68]. River-specific chromosomal profiles for the cytoforms of *S*. *colombaschense* reflect a pattern of dispersal along, rather than across, watercourses for oviposition. The same phenomenon has been reported in the *S*. *pictipes* group, which includes specialists of swift rivers with large rock outcrops [33]. Within the *S*. *tuberosum* group in North America, the species showing greatest site specificity, *S*. *tuberosum s*. *s*. (cytoform ‘AB’), inhabits larger streams and rivers than do other group members [35]. In contradistinction, habitat generalists, such as the pest species *P*. *mixtum* and *S*. *venustum*, show little, if any, site fidelity [69, 70].

## Conclusions

The advantages afforded by colonization of the largest rivers conspire to build pest populations that bankrupt the mind: 7 billion pupae of the North American cattle killer, *S*. *vampirum*, were estimated in one rocky weir across the North Saskatchewan River [71], and nearly 1 billion adults of *S*. *jenningsi* were estimated to emerge per km per day from large rivers in the eastern United States [62]. Populations of *S*. *vampirum* were estimated, before management, to be capable of withdrawing 2 liters of blood from every head of cattle in a 75,000-km^2^ area of Saskatchewan [72].

An understanding of the evolutionary factors that drive pest status can focus the search for genes responsible for the enabling traits. Such studies should begin with an assessment of biodiversity within the taxon of interest, sorting out populations, or species, hidden in the genome, which do not contribute to the pest problem, or perhaps are in danger of extinction, with consequent loss of ecosystem function [60]. The big-river specialist *S*. *colombaschense* provides a telling example of hidden taxonomic diversity and the associated differences in pest status, all masking as a single pest species. With the rapid progress in genomics of the Simuliidae [73], the genes associated with large-river specialization could be identified. Given structural and behavioral characters that facilitate the production of pests, coupled with an understanding of their underlying genetic basis, a new era of proactive pest management can be foreseen in which pest problems are predicted and prevented.
